# Ice sheets as a significant source of highly reactive nanoparticulate iron to the oceans

**DOI:** 10.1038/ncomms4929

**Published:** 2014-05-21

**Authors:** Jon R. Hawkings, Jemma L. Wadham, Martyn Tranter, Rob Raiswell, Liane G. Benning, Peter J. Statham, Andrew Tedstone, Peter Nienow, Katherine Lee, Jon Telling

**Affiliations:** 1 Bristol Glaciology Centre, School of Geographical Sciences, University of Bristol, University Road, Bristol BS8 1SS, UK; 2 Cohen Biogeochemistry Laboratory, School of Earth and Environment, University of Leeds, Leeds LS2 9JT, UK; 3 School of Ocean and Earth Science, National Oceanography Centre, University of Southampton, Southampton SO14 3ZH, UK; 4 School of Geoscience, University of Edinburgh, Edinburgh EH8 9XP, UK

## Abstract

The Greenland and Antarctic Ice Sheets cover ~\n10% of global land surface, but are rarely considered as active components of the global iron cycle. The ocean waters around both ice sheets harbour highly productive coastal ecosystems, many of which are iron limited. Measurements of iron concentrations in subglacial runoff from a large Greenland Ice Sheet catchment reveal the potential for globally significant export of labile iron fractions to the near-coastal euphotic zone. We estimate that the flux of bioavailable iron associated with glacial runoff is 0.40–2.54 Tg per year in Greenland and 0.06–0.17 Tg per year in Antarctica. Iron fluxes are dominated by a highly reactive and potentially bioavailable nanoparticulate suspended sediment fraction, similar to that identified in Antarctic icebergs. Estimates of labile iron fluxes in meltwater are comparable with aeolian dust fluxes to the oceans surrounding Greenland and Antarctica, and are similarly expected to increase in a warming climate with enhanced melting.

## Abstract (web summary)

Glacial meltwaters may help fertilize the iron-limited Polar Oceans, yet the contribution is poorly constrained. Hawkings *et al.* monitor iron fluxes during a full-melt season in Greenland, and propose that ice sheets provide highly reactive and potentially bioavailable iron, comparable with aeolian dust fluxes.

## Graphical abstract

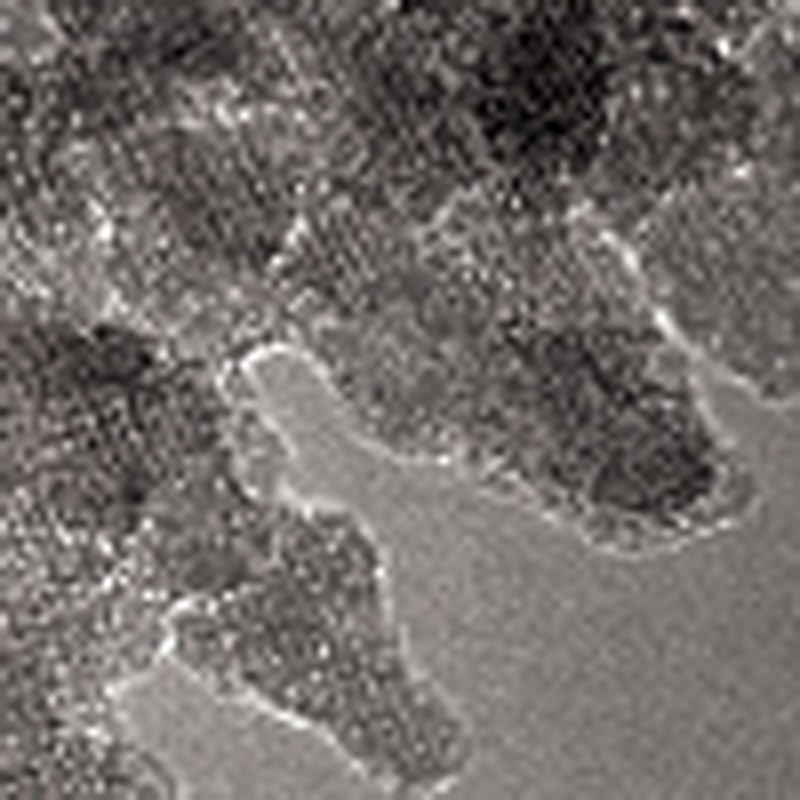

Iron limitation of primary producers is prevalent in large sectors of the world’s oceans, most notably the Southern Ocean (SO), the Pacific Northwest and minor parts of the North Atlantic (NA), all areas proximal to significant glacial activity^1^,^2^. These iron (Fe) limited oceans are believed to have an impact on the global climate as they play a role in regulating concentrations of atmospheric CO_2_ via their influence upon the strength of the biological pump^3^,^4^. Past studies of oceanic Fe inputs have focussed upon aeolian dust^5^,^6^, riverine discharge^7^, benthic recycling^7^ and sea ice^8^. Each source delivers Fe in a variety of phases, the solubilities (and bioavailabilities) of which are currently poorly understood, as are their transformations to more bioavailable forms^7^. More recently, ice sheets have also been hypothesized as contributors of iron to the oceans via icebergs^9^,^10^ and subglacial meltwaters^11^,^12^. Icebergs have been relatively well-studied in terms of their fertilization potential of the SO^9^,^10^,^13^, but both Fe concentrations and phase speciation in glacial runoff remain poorly constrained^14^. Glacial runoff accounts for around half of the freshwater exported from the Greenland Ice Sheet (GrIS) and recent observational evidence suggests that subglacial meltwaters are exported from the Antarctic Ice Sheet (AntIS) via channels beneath the margins of major ice streams^15^. We contend that a consideration of meltwater Fe fluxes, which supplements iron from icebergs, is critical for understanding oceanic iron cycling and primary productivity in polar waters^14^.

While there have been a few studies of Fe export in runoff from small valley glaciers in the Arctic^11^,^12^,^16^, there are currently no representive data on fluxes from large ice sheet catchments. There may be fundamental differences in subglacial chemical weathering between large ice sheet catchments and valley glaciers as a result of prolonged water residence times, greater anoxia and elevated physical erosion rates under the former^17^,^18^,^19^. Subglacial meltwater from the AntIS exits via ice streams, which terminate in large ice shelves, hindering direct sampling owing to logistical difficulties. The GrIS provides a more accessible ice sheet system, with large, land-terminating glaciers allowing direct sampling of subglacial waters at the ice margin. Additionally, meltwater stored over-winter at the bed is released episodically in summer via outburst events^20^. These present an opportunity to improve understanding of potential iron release during similar outbursts events observed around the continental margins in Antarctica^21^, linked to subglacial lake drainage events^22^,^23^, which may discharge large volumes of meltwater^24^.

Here we present the first dataset of iron fluxes from a large land-terminating catchment on the GrIS over a full-melt season, incorporating the release of long residence time waters from both early season runoff and subglacial outburst events. The underlying debris and morphology of the catchment is representative of >75% of the West Greenland ice margin^25^, and bedrock geology is predominantly Neoproterozoic gneiss/granitic, which is typical of large areas of the crystalline rocks that dominate the Precambrian Shield on which Greenland lies^26^. These data provide the most comprehensive, and therefore, most useful dataset to enable estimates of Fe input to polar waters. We propose that ice sheets provide a large and previously unconsidered source of highly reactive and potentially bioavailable subglacially derived iron to the Polar oceans, likely to sustain oceanic primary production.

## Results

### Iron phase speciation

Fe is known to exist in a spectrum of labile phases^7^, including ‘truly dissolved’ Fe (DFe), colloidal/nanoparticulate Fe (CNFe) and sediment-bound nanoparticulate Fe (SSFe), which fundamentally impact its subsequent transport and bioavailability. Hence, we measure two filterable phases in addition to the labile suspended sediment phase. Of the filterable phases, DFe is defined as Fe that passed through a 0.02 μm membrane, and CNFe is interpreted as that which passes through a 0.45 μm membrane minus DFe. SSFe is that extractable in an ascorbate solution, which preferentially dissolves labile (‘highly reactive’ ferrihydrite) iron, the most bioavailable form of iron oxyhydroxides^27^. These phases allow direct comparison with previous studies, by Statham *et al.*^11^ for DFe/CNFe, and the study by Raiswell *et al.*^10^ for SSFe.

**Figure 1:**
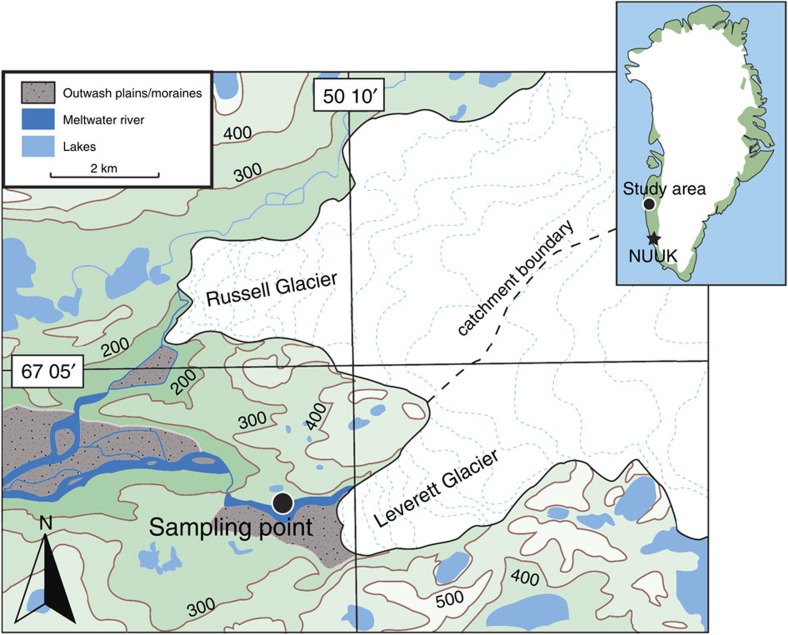
Location of Leverett catchment. A catchment boundary is shown, deduced from data published in Cowton et al.^19^ The glacier drains an area ~\n600 km^2^ of the Greenland Ice Sheet. Adapated from 1:100,000 map. The approximate sampling location is marked with a black dot in the main image.

**Figure 2:**
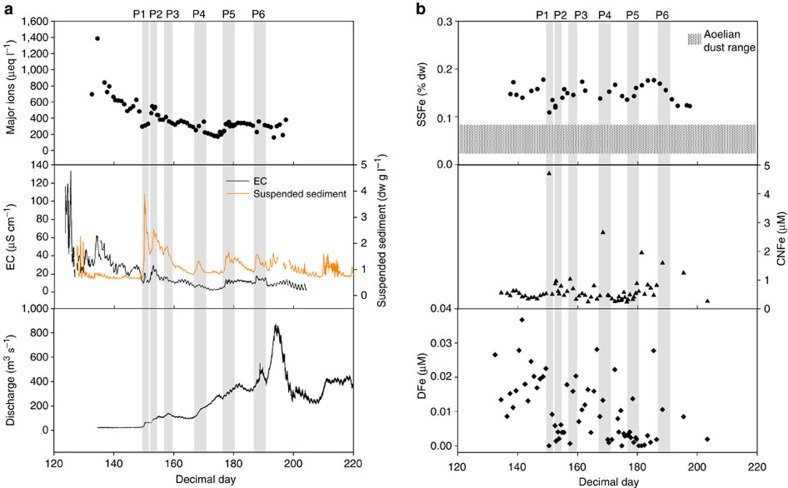
Time series from LG proglacial river. (**a**) Summed major ion concentration (K^+^, Na^+^,Ca^2+^,Mg^2+^, SO4^2−^, Cl^−^, HCO_3_^−^), Electrical conductivity (EC), suspended sediment concentrations and bulk discharge, and (**b**) of Fe fractions – SSFe, CNFe and DFe. The approximate timing of outburst events (P) is marked on **a** and **b** by shading. The range of ascorbate-extractable Fe concentrations found in aeolian dust is horizontally shaded in **b** for comparison.

Samples were collected at least once a day during the 2012 Greenland melt season from the subglacial channel draining Leverett Glacier (LG), South-West Greenland (Fig. 1). The glacier is ~\n85 km long, has a catchment of >600 km^2^ and mean summer discharge of >200 m^3^ s^*−*1^. It overlies predominantly Precambrian crystalline rocks, typical of large areas of Greenland. Over a melt season, the subglacial drainage system beneath LG evolves from a slow-inefficient drainage system to a fast-efficient channelized system^28^. Seasonal drainage evolution is accompanied by a number of substantial ‘outburst’ events (‘P’ events; Fig. 2), believed to be triggered by supra-glacial lake drainage events which force out solute enriched, long-term stored meltwater from the glacier bed^20^. Previous research indicates that these outburst events occur annually^19^,^20^.

**Figure 3:**
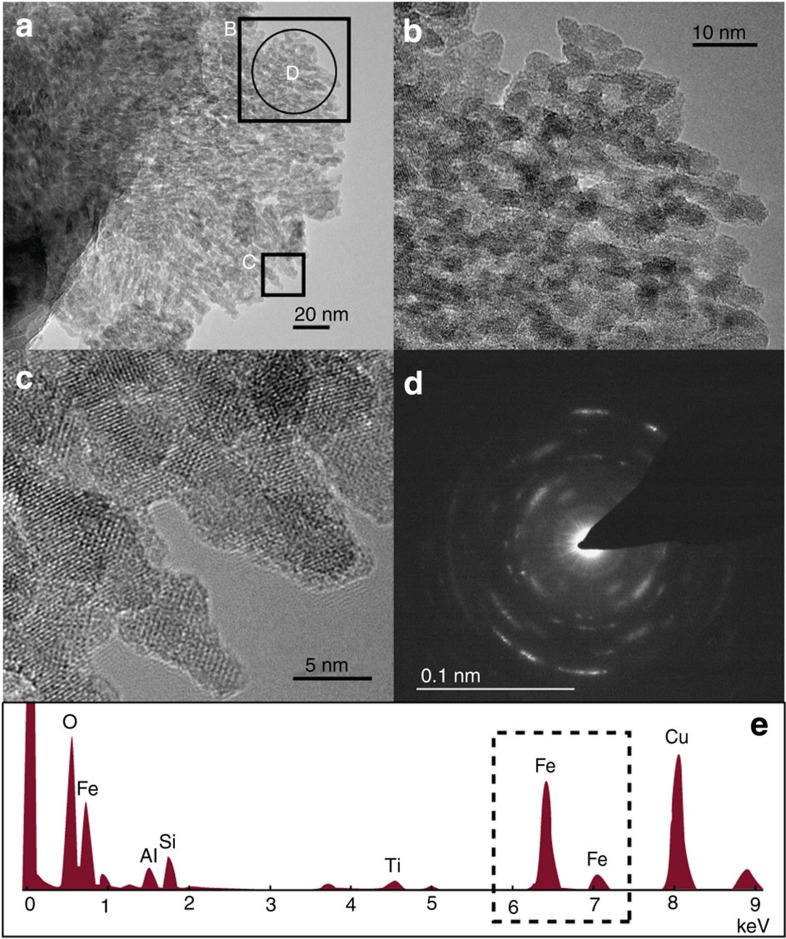
Photomicrographs of LG subglacial suspended sediment. Nanoparticulate ferrihyrite ~\n5–10 nm in diameter has been identified. Images (**b**) and (**c**) are enlargements of (**a**), as indicated. The diffraction signal (**d**) shows some crystalline structure owing to possible impact of nano-clay particles, and potentially nano-hematite, but also the characteristic diffuse ferrihydrite rings are identifiable. EDS (**e**) analysis of the area further confirms Fe-dominated material.

By far the most significant source of labile Fe in meltwaters was SSFe. Mean ascorbate-extractable Fe was 0.15% (dry weight; Table 1), equating to 29.0 μM of potentially bioavailable Fe. TEM microphotographs, spectral elemental analyses and nanodiffraction measurements of sediments confirmed that nanoparticulates were a mixture of clays and poorly ordered ferrihydrite around 5–10 nm diameter (Fig. 3). Ferrihydrite, a labile Fe nanoparticle, is indicative of recent Fe weathering and forms by the rapid oxidation of Fe^2+^ in solution. Fe^2+^ is expected to be generated in anoxic subglacial environments by microbially mediated sulphide oxidation^17^,^29^, and potentially via microbial Fe reduction^30^, although the latter is yet to be demonstrated.

The second most significant Fe phase is CNFe (Fig. 2b, middle). Discharge-weighted CNFe concentrations (mean Fe weighted for discharge at the time the sample was taken) were two orders of magnitude higher than DFe (699 nM; Table 1). Our CNFe concentrations spanned an order of magnitude, with a maximum concentration of 4,701 nM and a minimum concentration of 232 nM. The four highest measured CNFe concentrations were all closely associated with outburst events (Fig. 2; P1, P4, P5 and P6). High-suspended sediment loads were often associated with elevated CNFe concentrations (Fig. 2), as has been observed in riverine environments^31^.

In contrast to SSFe and CNFe, mean DFe concentrations in the bulk runoff were low (7 nM; Table 1), and comparable with studies of smaller catchments^11^. DFe concentrations generally decreased throughout the season as pH rose from ~\n7 to >9, and supra-glacial meltwater input increased (Fig. 2a). Higher early season concentrations of DFe are indicative of the release of concentrated meltwaters from distributed drainage systems, when supra-glacial input was low, as were suspended sediment concentrations.

### Ice sheet iron fluxes

To estimate an Fe budget for the entire GrIS we scale up our LG data (Fig. 2; Table 1). We use mean modelled runoff (418 km^3^ a^*−*1^) from 2000–2011 (ref. 32) as our representative runoff water flux, and modelled runoff (665 km^3^ a^*−*1^) for 2012^32^ as an indicator of possible future water fluxes in a warmer climate (2012 was a record melt year; Table 2)^32^,^33^. Based on 2000–2011 mean discharge, this generates a mean flux of 0.71 Tg Fe a^*−*1^ (0.40–2.54), of which 0.70 Tg (0.40–2.43) is SSFe, and 0.01 Tg (0.01–0.11) is DFe/CNFe (Table 2).

Antarctic subglacial waters are not diluted by supra-glacial meltwater. They may be anoxic, with rock: water contact times likely an order of magnitude greater than in Greenland^18^. As a result, solute concentrations have been measured in the millimolar range, compared with a micromolar range from the GrIS^18^,^34^. We therefore postulate that our Fe concentrations might be conservative estimates for the AntIS. Hence, we employ the maximum concentrations of DFe (0.04 μM) and CNFe (4.70 μM) from our dataset to calculate Antarctic fluxes (Table 2). This produces a filterable phase meltwater flux range of 8.6–25.8 Gg a^*−*1^, assuming a meltwater discharge of 32.5–97.5 km^3^ a^*−*1^ (ref. 35). Although little is known of suspended sediment concentrations in Antarctic subglacial meltwater, evidence exists in sub-marine core records^36^ for the release of sediment-rich meltwater plumes, and from first hand observations^21^. Using a lower order estimate of suspended sediment concentrations from Arctic glaciers (1 g l^*−*1^)^20^ and assuming an ascorbate-extractable fraction of 0.15%, the AntIS SSFe flux is 48.8–146.3 Gg a^−1^, which is of a similar order of magnitude to previous estimates^18^.

## Discussion

The data presented indicates that the potential for glacial iron export is large, driven primarily through SSFe and to a lesser extent CNFe. There appears to be no discernible trend in the SSFe concentrations through time (Fig. 2b, top: ±12.4%), suggesting subglacial sediment exported from the catchment is largely compositionally uniform. Ascorbate-extractable SSFe is significantly higher than values reported from aeolian dust (0.02–0.09%)^37^, and is close to those reported in Antarctic icebergs from Seymour Island (0.09–0.12%)^10^, and the Weddell Sea (0.04–0.4%; Table 1)^13^, indicating that glacial material is likely more bioavailable than aeolian dust. Culturing studies have already demonstrated the bioavailability of glacially derived sediment, with the addition of only 10 mg l^−1^ sediment increasing phytoplankton productivity in trace metal depleted waters^9^.

Despite being of less importance than SSFe, CNFe concentrations are more than an order of magnitude greater than those previously reported from a smaller Greenlandic catchment (~\n10 km in length), located ~\n30 km to the north in a catchment with a similar bedrock type^11^. This suggests that catchment size, and hence meltwater residence times, are important in determining CNFe concentrations where geology remains relatively uniform^17^. In comparison, a smaller catchment in Greenland, of different lithology, yielded mean CNFe values nearly an order of magnitude higher than those reported here^12^, indicating bedrock composition may be important in determining filterable iron concentrations. The periodic increase in the concentration of CNFe with outburst events (Fig. 2) is significant and suggests that the mode of subglacial meltwater release influences CNFe concentrations in meltwaters. It is hypothesized that dissolved (DFe) Fe(II) is formed *in situ* in isolated distributed systems within the subglacial environment, either in anoxic microcosms, or in more widespread anoxic systems^29^,^38^. Furthermore, observational evidence exists to suggest Fe(II) reservoirs may exist under the GrIS^39^,^40^. During outburst events (with elevated CNFe concentrations) Fe(II) from these more isolated parts of the drainage bed (long-term stored waters) may be oxidized *in situ* by injected O_2_ saturated supra-glacial waters. The exported CNFe may therefore partly reflect oxidation of a large-subglacial Fe(II) pool. This has implications when considering Antarctic subglacial Fe discharge, as there are no oxygenated supra-glacial inputs to the subglacial system^18^ and the ice sheet bed is thought to be anoxic^41^,^42^, suggesting that Fe released in meltwater may be predominantly as Fe(II). One recent study has demonstrated that particulate Fe in marine waters around the Antarctic coastline, near the Jutulstarmen ice stream, has distinctive Fe(II) dominated mineralogy^43^, which may suggest a subglacial source for the Fe(II). Hence, our data from outburst events at LG have clear relevance for Antarctica, where a substantial proportion of subglacial meltwater may be exported via subglacial lake drainage events^21^,^22^,^23^,^24^. Based upon these findings, we would anticipate Antarctic subglacial outbursts associated with lake drainage to also yield high concentrations of filterable Fe (CNFe+DFe).

DFe appears to be the least important component of glacial Fe export in bulk runoff. Although DFe concentrations rose during outburst events P5 and P6 (Fig. 2b), elevated levels were not consistent during all outburst events. Modification of DFe to CNFe fractions via oxidation and subsequent coagulation is likely to have occurred along the subglacial flowpath. Oxidation of DFe to CNFe (see discussion above) and eventual SSFe attachment, means that in Greenlandic catchments, DFe is likely less important than in Antarctica, where suboxic subglacial meltwater may directly enter oceanic waters^18^.

The glacial impact on ocean productivity in iron-limited areas will depend on the magnitude of the glacial flux and the bioavailability of the exported Fe. High rates of physical erosion beneath ice sheets^19^, combined with a suite of biogeochemical weathering processes that include sulphide oxidation^18^,^29^, indicates that ice sheets are a globally significant source of labile Fe nanoparticles. Given the representative bedrock type and large catchment size, we believe our values are typical of the large outlet glaciers, which dominate water fluxes from the GrIS^44^. Leverett catchment is more than an order of magnitude larger than previously studied Greenlandic catchments, with a mean discharge of 212 m^3^ s^−1^ (2009–2012) compared with ~\n15 m^3^ s^−1^ in the study by Statham *et al.*^11^ and <2 m^3^ s^−1^ in the study by Bhatia *et al.*^12^

The global significance of subglacial Fe depends not only on the mass delivered but also on its behaviour following deposition in seawater. This is true for all sources of Fe. Behaviour is complex; iron may be dissolved (inorganically, photochemically and/or by complexation) and can be precipitated or lost by aggregation, sinking and scavenging^7^. A detailed consideration of these effects is beyond the scope of the present paper and we therefore present only a simple flux comparison between potentially bioavailable Fe from subglacial sources, icebergs and aeolian dust.

The iron flux calculated for the GrIS is significant. It is greater than the estimated input of labile Fe nanoparticles into the NA by icebergs (0.25 Tg SSFe a^−1^, assuming similar reactivity and sediment content to AntIS icebergs)^7^, and comparable with aeolian dust input into the NA (0.04–0.16 Tg a^−1^, assuming Fe ascorbate solubility ranging from 0.02–0.085%)^5^,^37^, but with more localized input, and less efficient transport to the open ocean^45^. If we consider that only ~\n10% of Fe may reach the open ocean (that is, ~\n90% is removed in the estuarine zone and coastal waters)^7^, a conservative estimate of the GrIS meltwater Fe flux would be 0.07 Tg Fe a^−1^ (0.04–0.25), similar to aeolian dust input. We believe that the data presented here is a truly conservative estimate of the labile Fe flux from the GrIS to the surrounding ocean owing to a number of reasons: Leverett suspended sediment load has been reported to be higher in previous years, and as high as 10 g l^−1^ in other glacial catchments^19^; this does not include iron fluxes from Greenlandic icebergs (mean 497±50 km^3^ a^−1^ from 1958 to 2010)^44^, which have the potential for off-shelf fertilization; meltwater flux is predicted to increase in a warming climate (~\n60% higher based on preliminary 2012 GrIS meltwater flux; Table 1); and lastly, a number of Greenlandic glaciers discharge directly into the ocean^44^, avoiding estuarine processing^7^. There is also an increasing body of evidence for medium to long-range transport (~\n100–900 km) of Fe bearing particles away from shelf/terrestrial sources to HNLC waters, with subsequent interactions with biota^46^,^47^,^48^. However, unlike aeolian dust, medium to long-range transport may require the additional process of recycling from shelf sediments, and its effect is currently poorly understood. Although Fe limitation is not commonly observed in oceans around Greenland (potentially owing to subglacial and aeolian dust inputs) parts of the south-western coastal margin are postulated to be iron limited^49^,^50^,^51^, with large-annual blooms observed in this region strongly correlated to ice sheet meltwater input^52^.

Our estimated AntIS iron flux is significantly higher than the estimated flux of labile Fe from dust to the SO (5.3–23.0 Gg a^−1^, assuming Fe ascorbate solubility of 0.02–0.085%)^5^,^37^, although the input of meltwater-derived Fe will likely have a more localized impact than that of aeolian dust and iceberg-rafted Fe. Although meltwater fluxes of iron are an order of magnitude lower than the iceberg flux (600–1,200 Gg Fe a^−1^), recent work has demonstrated that subglacially derived Fe from ice streams may be able to travel up to 150 km offshore, fuelling productive phytoplankton blooms in the Amundsen Sea^46^. Significant lateral export of Fe, fuelling plankton blooms, has also been observed off other coastal areas of Antarctica^53^,^54^, and meltwater input has been correlated to large-annual phytoplankton blooms off the Antarctic Peninsula, located both locally to the source location and over 100 km offshore^55^. Furthermore, a recent study found a unique Fe(II) signature downstream of the Jutulstraumen Ice Stream in Antarctica^43^. However, the source and mechanism of delivery wasn’t known and the link to glacial input of bioavailable Fe is yet to be firmly established.

We conclude that ice sheets are likely to play a more significant role in the global iron cycle than previously recognized, via fresh subglacial weathering of Fe bearing minerals. SSFe and CNFe fluxes from the Antarctic and Greenland Ice Sheets are comparable, if not larger, than aeolian dust input to their respective regions (NA and SO), but may have a more localized impact owing to point source input. Our iron flux estimates for the AntIS demonstrate that meltwater discharge may supplement bioavailable Fe delivery to the SO from icebergs and aeolian dust, and thus, should be considered in future climate models. The impact of global warming on these iron budgets is unknown. However, it is likely that ice sheets will provide a greater flux of bioavailable Fe to coastal regions as larger quantities of meltwater are exported to the oceans in a warmer climate.

## Methods

### Water sample collection and filtration

Bulk meltwater samples were taken throughout main melt period (May June and July 2012) around ~\n1 km downstream from the glacier terminus (Fig. 1). For the majority of the sampling period, samples were collected at least once a day, always at 10:00 h, and on occasion at 18:00 h to observe diurnal variation. We know these waters were representative of bulk discharge as LG drains from a single portal on the northern side of the terminus^20^, and point samples taken at the portal were of similar concentration to those taken downstream. Suspended sediment samples (SSFe) were collected by filtering ~\n200 ml of meltwater onto a 0.45 μm cellulose nitrate filter (Whatman). In accordance with studies of iron (oxy)hydroxide stability^7^,^27^,^56^ samples were stored damp on the filter papers in a refrigerated 25 ml polypropylene bottle until analysis at the University of Leeds, ~\n3–4 months after collection. Little aging of freshly precipitated (oxy)hydroxides was therefore expected as the half life of transformation to more crystalline forms is >500 days at this temperature.

DFe and CNFe were collected according to trace metal procedures adopted by Schroth *et al.*^16^, from methods developed by Shiller^31^, and by using the trace metal sampling practices of Howard and Statham^57^, with subsequent improvements for glacial meltwaters (Statham, personal communication). Briefly, all sampling equipment was sequentially soaked in a 6 M HCl acid bath (24 h), washed × 3 with ultrapure 18.2 MΩ cm Milli-Q water (Millipore), soaked in a 6 M HNO_3_ acid bath (24 h), with a final × 6 wash with Milli-Q water before drying in a laminar flow hood. All sampling bottles were trace metal grade Nalgene LDPE (Thermo Scientific). Filters were cleaned with trace metal grade HCl in accordance with the study by Shiller^31^. CNFe was defined by using a 0.45 μm Whatman GD/XP syringe filter with polypropylene pre-filters and a polyethersulfone final filter (designed for trace metal analysis, high-particulate load samples). DFe was defined by using Whatman Anotop 0.02 μm syringe filters. Samples filtered for 0.02 μm used a 0.45 μm GD/XP filter as a pre-filter. Filtration was always conducted in a designated ‘clean’ lab tent, within a labmade box (low-density polyethylene sheet plastic covering a polycarbonate piping frame), thus minimizing any contamination with dust. Samples were preserved in the field by acidifying with Optima HNO_3_ (Fisher) to a pH<2. Field procedural blanks were taken using transported Milli-Q water, using the same procedures that had been applied to glacial samples.

### SSFe extractions

Sediment extractions were carried out according to the study by Raiswell *et al.*^27^, with sequential extractions for amorphous ferrihydrite (ascorbate), and crystalline Fe (oxy)hydroxides (dithionite–data not presented in this study). Total Fe was analyzed by atomic absorption spectroscopy using an Analytik Jena High-Resolution Continuum Source, ContrAA 700 instrument at the School of Earth and Environment, University of Leeds. A procedural blank was below the detection limit of the instrument, while replicates showed a standard deviation of ±1.5%.

### DFe and CNFe determination

DFe and CNFe were determined at the National Oceanography Centre (Southampton) Mass Spectrometer Lab, using a Thermo Scientific XSERIES 2 quadrupole ICP-MS, with Be, In and Re as internal standards. CNFe blanks were 1.2±0.7% of the lowest recorded concentration. DFe blanks were at or below the machine’s detection limit of ~\n1 nM—sample values lower than this were recorded as <d1.

### Microspectroscopic and nanodiffraction analyses

The morphology, structure and crystallinity of all phases but with particular focus on Fe (oxy)hydroxides were determined using Field Emission Gun Transmission Electron Microscopy (FEG-TEM; Tecnai) operating at 200 kV. Samples were dispersed in ethanol using an ultrasonic bath for ~\n1 min, and then a drop was pipetted onto an Agar standard holey carbon support films. Low fluency and high-resolution images of nanoparticles were complemented by energy dispersive spectra (acquired with an Oxford Instrument EDS analyses system) and selected area electron diffraction patterns that were recorded to determine elemental characteristics of identified Fe nanophases.

### Mass flux for leverett catchment

Total-meltwater flux from Leverett catchment was calculated from a season long record of meltwater discharge as in the study by Cowton *et al.*^19^ (Fig. 2). Briefly, the meltwater river was monitored in a stable bedrock section ~\n2.2 km downstream of the terminus. Stage was logged every 5 min until July, when it was logged every 10 min. This was converted to discharge using a rating curve of rhodamine dye-dilution experiments.

Suspended sediment flux was calculated from suspended sediment concentrations^19^, which were multiplied by discharge at each logged time point. Suspended sediment concentration was derived from a season long logged turbidity sensor. The turbidity sensor was calibrated using manual sediment collections. Briefly, 300 ml of meltwater was filtered through a 0.45 μm cellulose nitrate filter, oven dried overnight at 40 °C and weighed. Suspended sediment flux was calculated from the combined discharge and suspended sediment concentration time series.

### Mass fluxes for the greenland and AntISs

Mean modelled GrIS runoff from the study by Tedesco *et al.*^32^ for the period 2000–2011 is used, alongside the modelled 2012 runoff, a record melt year that may provide an indication of future meltwater flux. Suspended sediment flux was calculated from minimum (0.643 g l^−1^) discharge-weighted mean (1.109 g l^−1^) and maximum (3.876 g l^−1^) recorded concentrations from Leverett catchment, multiplied by the Greenland meltwater flux.

For Antarctic meltwater flux, modelled basal melt rates from the study by Pattyn^35^, of 65 km^3^, with a standard deviation of ±50% for minimum and maximum estimates were used to calculate meltwater flux from the Ice Sheet. Suspended sediment flux was estimated using a 1 g l^−1^ suspended sediment concentration, as in the study by Wadham *et al.*^18^

## Author contributions

All authors made significant contributions to the research presented here. J.L.W. and M.T. conceived the project. J.R.H., R.R., A.T., P.N., K.L., J.T. and J.L.W. collected the field data. J.R.H. and J.L.W. wrote the manuscript. J.R.H. undertook lab analysis. L.G.B. provided significant help and invaluable advice in lab analysis. P.S. and R.R. aided in developing the field methods.

## Additional information

**How to cite this article:** Hawkings, J. R. *et al.* Ice sheets as a significant source of highly reactive nanoparticulate iron to the oceans. *Nat. Commun.* 5:3929 doi: 10.1038/ncomms4929 (2014).

## Figures and Tables

**Table 1:** Meltwater and suspended sediment Fe concentrations from Leverett Glacier and comparative studies.

**Fe source**	**Mean Fe concentrations**	**s.d.**	**Fe range**	**n**	**Source**
*Filtered iron source (nM)*					
Greenland					
<0.02 μm	7	9	<d1–37	66	DFe—this study
0.02–0.45 μm	699	650	232–4,701	63	CNFe—this study
<0.03 μm—GrIS	22		6–59	15	11
0.03–0.4 μm—GrIS	30.8		2–117	15	11
<0.2 μm—GrIS	3,700		2,200–9,310	13	12
					
Antarctica					
<0.2 μm—Blood Falls, AntIS	4 × 10^6^			1	41
<0.45 μm—Dry Valleys, AntIS	335		82–1,146	11	58

*Ascorbate-extractable sediments (% dw)*					
Glacial meltwater—GrIS	0.15	0.02	0.11–0.18	33	SSFe—this study
Icebergs—AntIS	0.15	0.12	0.06–0.36	10	10
Icebergs—AntIS	0.19	0.18	0.04–0.49	4	13
Aoelian dust—East Med.	0.03			1	37
Aoelian dust—West Med.	0.08			1	37
Aoelian dust—Sahara	0.02			2	37
Aoelian dust—Sahel	0.02			2	37
Aoelian dust—Beijing	0.06			1	37

AntIS, Antarctic ice sheet; CNFe, colloidal/nanoparticulate Fe; DFe, dissolved Fe; GrIS, Greenland ice sheet.

Filtered concentrations are discharge weighted and presented in nanomolar. Values below the detection limit are indicated by <d1. Ascorbate-extractable concentrations are presented as % dry weight (% dw).

**Table 2:** Fe fluxes from Leverett Glacier and scaled up estimates for the Greenland Ice Sheet and Antarctic Ice Sheet.

**Source**	**Mass flux**	**Fe flux a**^**−1**^		
		**Minimum**	**Mean**	**Maximum**
*Leverett catchment (Mg)*				
DFe*	2.20 km^3^ a^−1^	0.0	0.9	4.5
CNFe*	2.20 km^3^ a^−1^	28.5	85.8	578
SSFe†	2.31 Tg a^−1^	2,540	3,470	4,160
Total labile Fe		2,570	3,560	4,740
				
*Greenland Ice Sheet (Gg)*				
2000–2011 mean Q				
DFe‡	418 km^3^ a^−1^	0.0	0.2	0.9
CNFe‡	418 km^3^ a^−1^	5.4	16.3	110
SSFe§	0.3–1.6 Pg a^−1^	403	695	2,430
Total labile Fe		409	712	2,540
				
*2012 Q (Gg)*				
DFe‡	665 km^3^ a^−1^	0.0	0.3	1.4
CNFe‡	665 km^3^ a^−1^	8.6	26.0	175
SSFe§	0.4–2.6 Pg a^−1^	642	1,110	3,870
Total labile Fe		651	1,140	4,050
				
*Antarctic Ice Sheet (Gg)*				
DFe||	32.5–97.5 km^3^ a^−1^	0.1	0.1	0.2
CNFe||	32.5–97.5 km^3^ a^−1^	8.5	17.1	25.6
SSFe¶	0.03–0.10 Pg a^−1^	48.8	97.5	146
Iceberg Fe#	1.25 Pg a^−1^	600	900	1,200
Total labile Fe		657	1,010	1,370

CNFe, colloidal/nanoparticulate Fe; DFe, dissolved Fe; SSFe, sediment-bound nanoparticulate Fe.

Results presented to three significant figures.

Q indicates meltwater discharge.

^*^Calculated from minimum discharge-weighted mean and maximum Fe concentrations multiplied by the Leverett meltwater flux.

^†^Calculated using minimum (0.11%) discharge-weighted mean (0.15%) and maximum (0.18%) FeA concentrations, multiplied by the suspended sediment flux.

^‡^Calculated from minimum discharge-weighted mean and maximum recorded concentrations from Leverett catchment, multiplied by the Greenland meltwater flux.

^§^Calculated using minimum (0.643 g l^−1^), discharge-weighted mean (1.109 g l^−1^) and maximum (3.876 g l^−1^) suspended sediment concentrations recorded from Leverett catchment, and the discharge weight mean Leverett FeA of 0.15%. We decided to use this method of calculation owing to the uncertainty surrounding mean suspended sediment concentrations in Greenlandic meltwaters, compared with the low standard deviation of FeA in samples.

^||^Estimated using minimum (50% of mean), mean and maximum (150% of mean) meltwater fluxes from the study by Pattyn^35^ coupled with maximum DFe and CNFe concentrations recorded from Leverett catchment.

^¶^Estimated using minimum (50% of mean), mean and maximum (150% of mean) meltwater fluxes from the study by Pattyn^35^, a suspended sediment load of 1 g l^−1^ and mean Leverett FeA of 0.15%.

^#^From the study by Raiswell *et al.*^10^

## References

[b1] Martin J. H., Fitzwater S. E. & Gordon R. M. Iron deficiency limits phytoplankton growth in Antarctic waters. Glob. Biogeochem. Cycles 4, 5–12 (1990).

[b2] Nielsdottir M. C., Moore C. M., Sanders R., Hinz D. J. & Achterberg E. P. Iron limitation of the postbloom phytoplankton communities in the Iceland Basin. Glob. Biogeochem. Cycles 23, GB3001 (2009).

[b3] de Baar H. J. W., Gerringa L. J. A., Laan P. & Timmermans K. R. Efficiency of carbon removal per added iron in ocean iron fertilization. Mar. Ecol. Prog. Ser. 364, 269–282 (2008).

[b4] Laws E. A., Falkowski P. G., Smith W. O., Ducklow H. & McCarthy J. J. Temperature effects on export production in the open ocean. Glob. Biogeochem. Cycles 14, 1231–1246 (2000).

[b5] Jickells T. D. *et al.* Global iron connections between desert dust, ocean biogeochemistry, and climate. Science 308, 67–71 (2005). 15802595 10.1126/science.1105959

[b6] Fan S. M., Moxim W. J. & Levy H. Aeolian input of bioavailable iron to the ocean. Geophys. Res. Lett. 33, L07602 (2006).

[b7] Raiswell R. & Canfield D. E. The iron biogeochemical cycle past and present. Geochem. Perspect. 1, 1–220 (2012).

[b8] Lannuzel D., Schoemann V., de Jong J., Tison J. L. & Chou L. Distribution and biogeochemical behaviour of iron in the East Antarctic sea ice. Mar. Chem. 106, 18–32 (2007).

[b9] Smith K. L. *et al.* Free-drifting icebergs: Hot spots of chemical and biological enrichment in the Weddell Sea. Science 317, 478–482 (2007). 17588896 10.1126/science.1142834

[b10] Raiswell R., Benning L. G., Tranter M. & Tulaczyk S. Bioavailable iron in the Southern Ocean: the significance of the iceberg conveyor belt. Geochem. Trans. 9, 7 (2008). 18513396 10.1186/1467-4866-9-7 PMC2440735

[b11] Statham P. J., Skidmore M. & Tranter M. Inputs of glacially derived dissolved and colloidal iron to the coastal ocean and implications for primary productivity. Glob. Biogeochem. Cycles 22, GB3013 (2008).

[b12] Bhatia M. P. *et al.* Greenland meltwater as a significant and potentially bioavailable source of iron to the ocean. Nat. Geosci. 6, 274–278 (2013).

[b13] Shaw T. J. *et al.* Input, composition, and potential impact of terrigenous material from free-drifting icebergs in the Weddell Sea. Deep-Sea Res. II 58, 1376–1383 (2011).

[b14] Death R. *et al.* Antarctic Ice Sheet fertilises the Southern Ocean. Biogeosciences Discuss. 10, 12551–12570 (2013).

[b15] Le Brocq A. *et al.* Evidence from ice shelves for channelized meltwater flow beneath the Antarctic Ice Sheet. Nat. Geosci. 6, 945–948 (2013).

[b16] Schroth A. W., Crusius J., Chever F., Bostick B. C. & Rouxel O. J. Glacial influence on the geochemistry of riverine iron fluxes to the Gulf of Alaska and effects of deglaciation. Geophys. Res. Lett. 38, L16605 (2011).

[b17] Wadham J. L. *et al.* Biogeochemical weathering under ice: size matters. Glob. Biogeochem. Cycles 24, GB3025 (2010).

[b18] Wadham J. *et al.* The potential role of the Antarctic Ice Sheet in global biogeochemical cycles. Earth Environ. Sci. Trans. R. Soc. Edinb. 104, 55–67 (2013).

[b19] Cowton T., Nienow P., Bartholomew I., Sole A. & Mair D. Rapid erosion beneath the Greenland ice sheet. Geology 40, 343–346 (2012).

[b20] Bartholomew I. *et al.* Supraglacial forcing of subglacial drainage in the ablation zone of the Greenland ice sheet. Geophys. Res. Lett. 38, L08502 (2011).

[b21] Goodwin L. The nature and origin of a jokulhlaup near Casey Station, Antarctica. J. Glaciol. 34, 95–101 (1988).

[b22] Wingham D. J., Siegert M. J., Shepherd A. & Muir A. S. Rapid discharge connects Antarctic subglacial lakes. Nature 440, 1033–1036 (2006). 16625193 10.1038/nature04660

[b23] Fricker H. A., Scambos T., Bindschadler R. & Padman L. An active subglacial water system in West Antarctica mapped from space. Science 315, 1544–1548 (2007). 17303716 10.1126/science.1136897

[b24] McMillan M. *et al.* Three-dimensional mapping by CryoSat-2 of subglacial lake volume changes. Geophys. Res. Lett. 40, 4321–4327 (2013).

[b25] Knight P. G., Waller R. I., Patterson C. J., Jones A. P. & Robinson Z. P. Discharge of debris from ice at the margin of the Greenland ice sheet. J. Glaciol. 48, 192–198 (2002).

[b26] Kalsbeek F. The evolution of the Precambrian Shield of Greenland. Geol. Rundsch. 71, 38–60 (1982).

[b27] Raiswell R., Vu H. P., Brinza L. & Benning L. G. The determination of labile Fe in ferrihydrite by ascorbic acid extraction: methodology, dissolution kinetics and loss of solubility with age and de-watering. Chem. Geol. 278, 70–79 (2010).

[b28] Chandler D. M. *et al.* Evolution of the subglacial drainage system beneath the Greenland Ice Sheet revealed by tracers. Nat. Geosci. 6, 195–198 (2013).

[b29] Tranter M., Sharp M., Lamb H., Brown G., Hubbard B. & Willis I. Geochemical weathering at the bed of Haut Glacier d'Arolla, Switzerland, a new model. Hydrol. Process. 16, 959–993 (2002).

[b30] Lovley D. R. Dissimilatory Fe(III) and Mn(IV) reduction. Microbiol. Rev. 55, 259–287 (1991). 1886521 10.1128/mr.55.2.259-287.1991 PMC372814

[b31] Shiller A. M. Syringe filtration methods for examining dissolved and colloidal trace element distributions in remote field locations. Environ. Sci. Technol. 37, 3953–3957 (2003). 12967118 10.1021/es0341182

[b32] Tedesco M. *et al.* Evidence and analysis of 2012 Greenland records from spaceborne observations, a regional climate model and reanalysis data. Cryosphere 7, 615–630 (2013).

[b33] Nghiem S. V. *et al.* The extreme melt across the Greenland ice sheet in 2012. Geophys. Res. Lett. 39, L20502 (2012).

[b34] Skidmore M., Tranter M., Tulaczyk S. & Lanoil B. Hydrochemistry of ice stream beds - evaporitic or microbial effects? Hydrol. Process. 24, 517–523 (2010).

[b35] Pattyn F. Antarctic subglacial conditions inferred from a hybrid ice sheet/ice stream model. Earth Planet. Sci. Lett. 295, 451–461 (2010).

[b36] Lowe A. L. & Anderson J. B. Reconstruction of the West Antarctic Ice Sheet in Pine Island Bay during the last glacial maximum and its subsequent retreat history. Quat. Sci. Rev. 21, 1879–1897 (2002).

[b37] Shi Z. B. *et al.* Impacts on iron solubility in the mineral dust by processes in the source region and the atmosphere: A review. Aeolian Res. 5, 21–42 (2012).

[b38] Bottrell S. H. & Tranter M. Sulphide oxidation under partially anoxic conditions at the bed of the Haut Glacier d'Arolla, Switzerland. Hydrol. Process. 16, 2363–2368 (2002).

[b39] Yde J. C. *et al.* Basal ice microbiology at the margin of the Greenland ice sheet. Ann. Glaciol. 51, 71–79 (2010).

[b40] Christner B. C., Montross G. G. & Priscu J. C. Dissolved gases in frozen basal water from the NGRIP borehole: implications for biogeochemical processes beneath the Greenland Ice Sheet. Polar Biol. 35, 1735–1741 (2012).

[b41] Mikucki J. A. *et al.* A contemporary microbially maintained subglacial ferrous ‘ocean’. Science 324, 397–400 (2009). 19372431 10.1126/science.1167350

[b42] Wadham J. L. *et al.* Potential methane reservoirs beneath Antarctica. Nature 488, 633–637 (2012). 22932387 10.1038/nature11374

[b43] von der Heyden B. P., Roychoudhury A. N., Mtshali T. N., Tyliszczak T. & Myneni S. C. B. Chemically and geographically distinct solid-phase iron pools in the Southern Ocean. Science 338, 1199–1201 (2012). 23197531 10.1126/science.1227504

[b44] Bamber J., van den Broeke M., Ettema J., Lenaerts J. & Rignot E. Recent large increases in freshwater fluxes from Greenland into the North Atlantic. Geophys. Res. Lett. 39, L19501 (2012).

[b45] Li F., Ginoux P. & Ramaswamy V. Distribution, transport, and deposition of mineral dust in the Southern Ocean and Antarctica: contribution of major sources. J. Geophys. Res. Atmos. 113, D10207 (2008).

[b46] Gerringa L. J. A. *et al.* Iron from melting glaciers fuels the phytoplankton blooms in Amundsen Sea (Southern Ocean): iron biogeochemistry. Deep-Sea Res. II 71-76, 16–31 (2012).

[b47] Lam P. J. *et al.* Wintertime phytoplankton bloom in the subarctic Pacific supported by continental margin iron. Glob. Biogeochem. Cycles 20, GB1006 (2006).

[b48] Planquette H., Sanders R. R., Statham P. J., Morris P. J. & Fones G. R. Fluxes of particulate iron from the upper ocean around the Crozet Islands: A naturally iron-fertilized environment in the Southern Ocean. Glob. Biogeochem. Cycles 25, GB2011 (2011).

[b49] Tagliabue A., Bopp L. & Aumont O. Ocean biogeochemistry exhibits contrasting responses to a large scale reduction in dust deposition. Biogeosciences 5, 11–24 (2008).

[b50] Moore J. K., Doney S. C., Glover D. M. & Fung I. Y. Iron cycling and nutrient-limitation patterns in surface waters of the World Ocean. Deep-Sea Res. II 49, 463–507 (2002).

[b51] Saba V. S. *et al.* An evaluation of ocean color model estimates of marine primary productivity in coastal and pelagic regions across the globe. Biogeosciences 8, 489–503 (2011).

[b52] Frajka-Williams E. & Rhines P. B. Physical controls and interannual variability of the Labrador Sea spring phytoplankton bloom in distinct regions. Deep-Sea Res. II 57, 541–552 (2010).

[b53] Charette M. A. *et al.* Radium isotopes as tracers of iron sources fueling a Southern Ocean phytoplankton bloom. Deep-Sea Res. II 54, 1989–1998 (2007).

[b54] Dulaiova H., Ardelan M. V., Henderson P. B. & Charette M. A. Shelf-derived iron inputs drive biological productivity in the southern Drake Passage. Glob. Biogeochem. Cycles 23, GB4014 (2009).

[b55] Dierssen H. M., Smith R. C. & Vernet M. Glacial meltwater dynamics in coastal waters west of the Antarctic peninsula. Proc. Natl Acad. Sci. USA 99, 1790–1795 (2002). 11830636 10.1073/pnas.032206999 PMC122272

[b56] Schwertmann U., Stanjek H. & Becher H. H. Long-term *in vitro* transformation of 2-line ferrihydrite to goethite/hematite at 4, 10, 15 and 25 degrees C. Clay Miner. 39, 433–438 (2004).

[b57] Howard A. G. & Statham P. Inorganic Trace Analysis: Philosophy and Practice Wiley (1993).

[b58] Green W. J., Stage B. R., Preston A., Wagers S., Shacat J. & Newell S. Geochemical processes in the Onyx River, Wright Valley, Antarctica: major ions, nutrients, trace metals. Geochim. Cosmochim. Acta 69, 839–850 (2005).

